# Supplementary material for: STAG2 loss in Ewing sarcoma alters enhancer-promoter contacts dependent and independent of EWS::FLI1
Source: EMBO Rep. 2024 Nov 1;25(12):5537–60. doi: 10.1038/s44319-024-00303-6 (PMC11624272; doi:10.1038/s44319-024-00303-6)
Supplement: Supplementary file 3 — Appendix [file 44319_2024_303_MOESM3_ESM.pdf]

Appendix

STAG2 loss in Ewing sarcoma alters enhancer-promoter contacts dependent and independent of EWS::FLI1

Daniel Giménez-Llorente[1], Ana Cuadrado[1#], María José Andreu[1], Inmaculada Sanclemente-Alamán[1], Maria Solé-Ferran[1], Miriam Rodríguez-Corsino[1] and Ana Losada[1#]

Table of contents

1. Appendix Figure S1……..page 2
2. Appendix Figure S2……..page 3
3. Appendix Figure S3……..page 4
4. Appendix Figure S4……..page 5

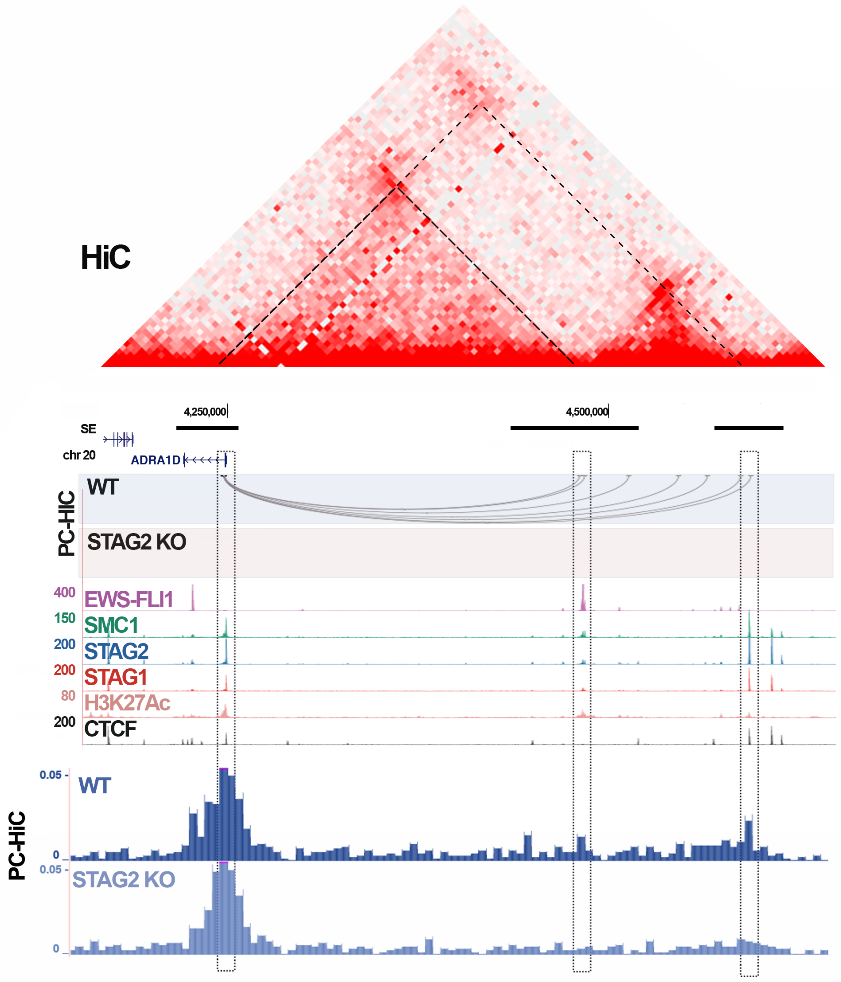

Appendix Figure S1. Differential contacts from the *ADRA1D* gene promoter
Genomic landscape of the region encompassing the *ADRA1D* gene, as in Figure 4G, to which we have added "virtual 4C" tracks displaying the interaction frequency of the gene promoter with neighboring regions in STAG2 WT and KO conditions.

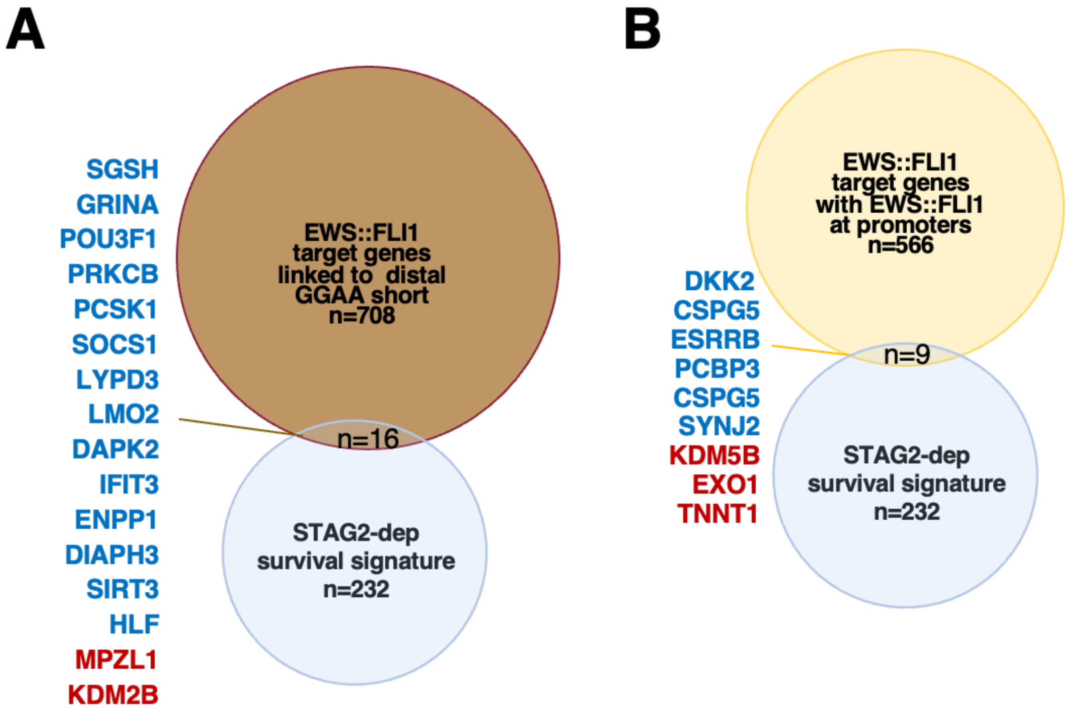

Appendix Figure S2. Effect of STAG2 loss on EWS::FLI1 target genes
Venn diagrams displaying the overlap between genes deregulated in Ewing sarcoma cells and patients without STAG2 and EWS::FLI1 target genes whose promoters are either linked to EWS::FLI1-bound GGAA(1-4) repeat regions (A) or directly bound by the oncoprotein (B). Downregulated and upregulated genes appear in blue and red, respectively.

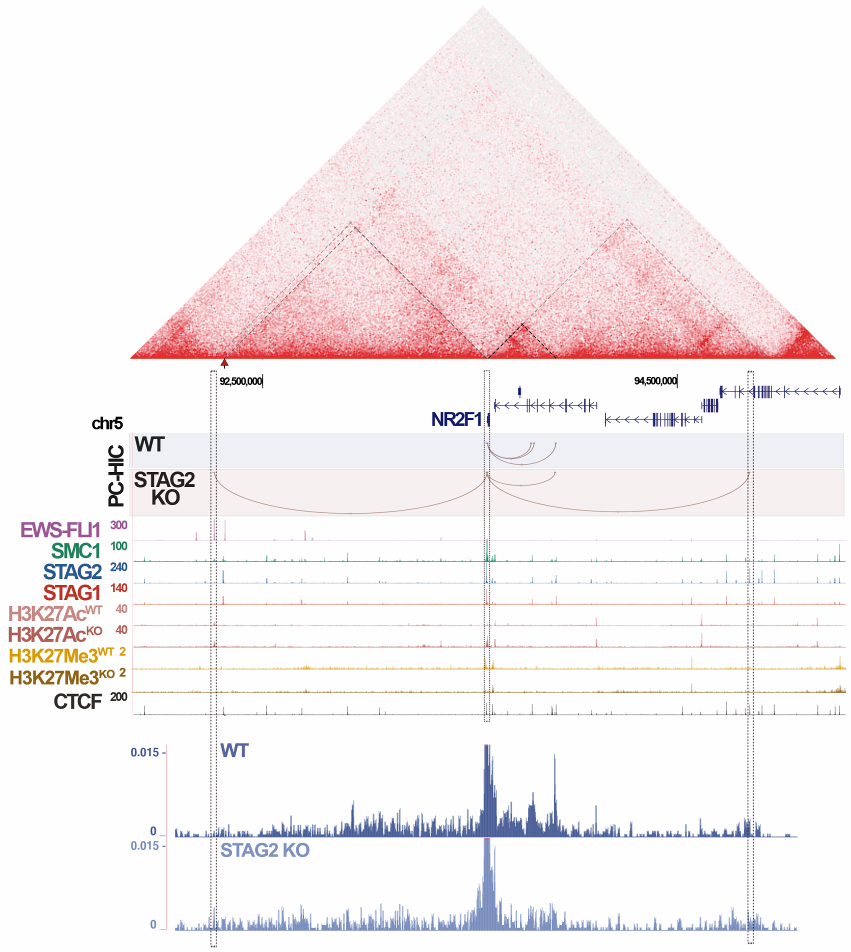

Appendix Figure S3. Differential contacts from the *NR2F1* gene promoter
Genomic landscape of the region encompassing the *NR2F1* gene, as in Figure 5A, to which we have added "virtual 4C" tracks displaying the interaction frequency of the gene promoter with neighboring regions in *STAG2* WT and KO conditions.

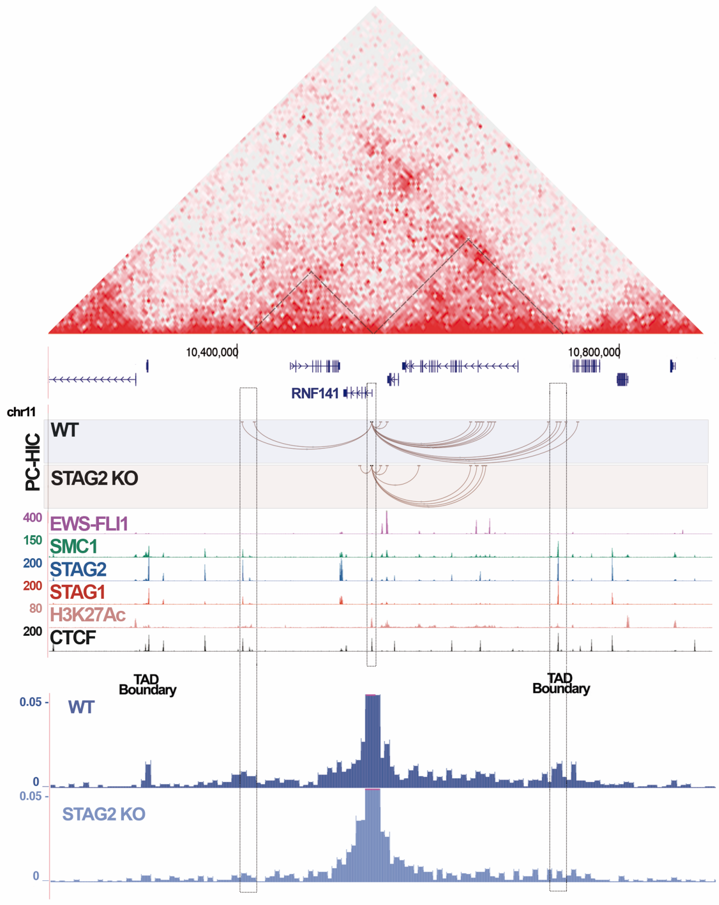

Appendix Figure S4. Differential contacts from the *RNF141* gene promoter
Genomic landscape of the region encompassing the *RNF141* gene, as in Figure 6D, to which we have added "virtual 4C" tracks displaying the interaction frequency of the gene promoter with neighboring regions in *STAG2* WT and KO conditions.
