# Supplementary figures and images for: STAG2 loss in Ewing sarcoma alters enhancer-promoter contacts dependent and independent of EWS::FLI1
Source: EMBO Rep. 2024 Nov 1;25(12):5537–60. doi: 10.1038/s44319-024-00303-6 (PMC11624272; doi:10.1038/s44319-024-00303-6)

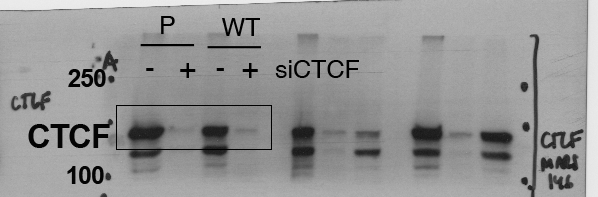

Supplement: Supplementary file 12 — Source data Fig. 5 [file 44319_2024_303_MOESM12_ESM.zip › 5C/western CTCF.tif]

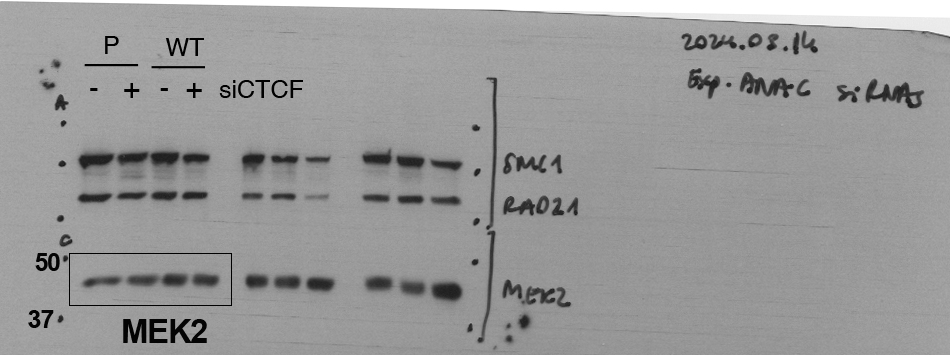

Supplement: Supplementary file 12 — Source data Fig. 5 [file 44319_2024_303_MOESM12_ESM.zip › 5C/western MEK2.tif]

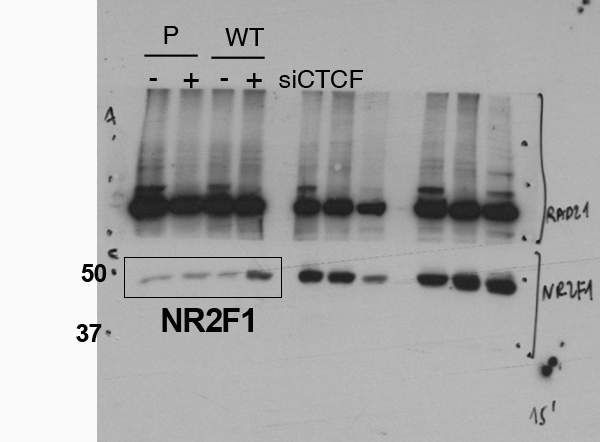

Supplement: Supplementary file 12 — Source data Fig. 5 [file 44319_2024_303_MOESM12_ESM.zip › 5C/western NR2F1.tif]

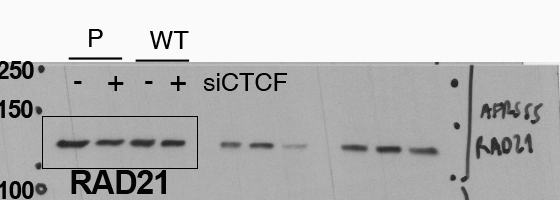

Supplement: Supplementary file 12 — Source data Fig. 5 [file 44319_2024_303_MOESM12_ESM.zip › 5C/western RAD21.tif]

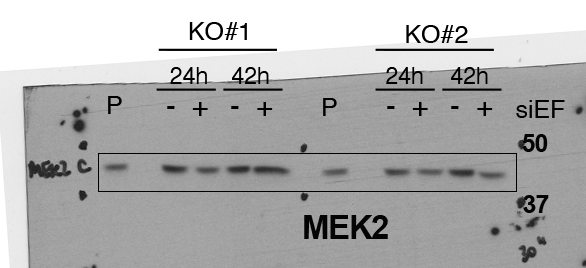

Supplement: Supplementary file 12 — Source data Fig. 5 [file 44319_2024_303_MOESM12_ESM.zip › 5B/western MEK2.tif]

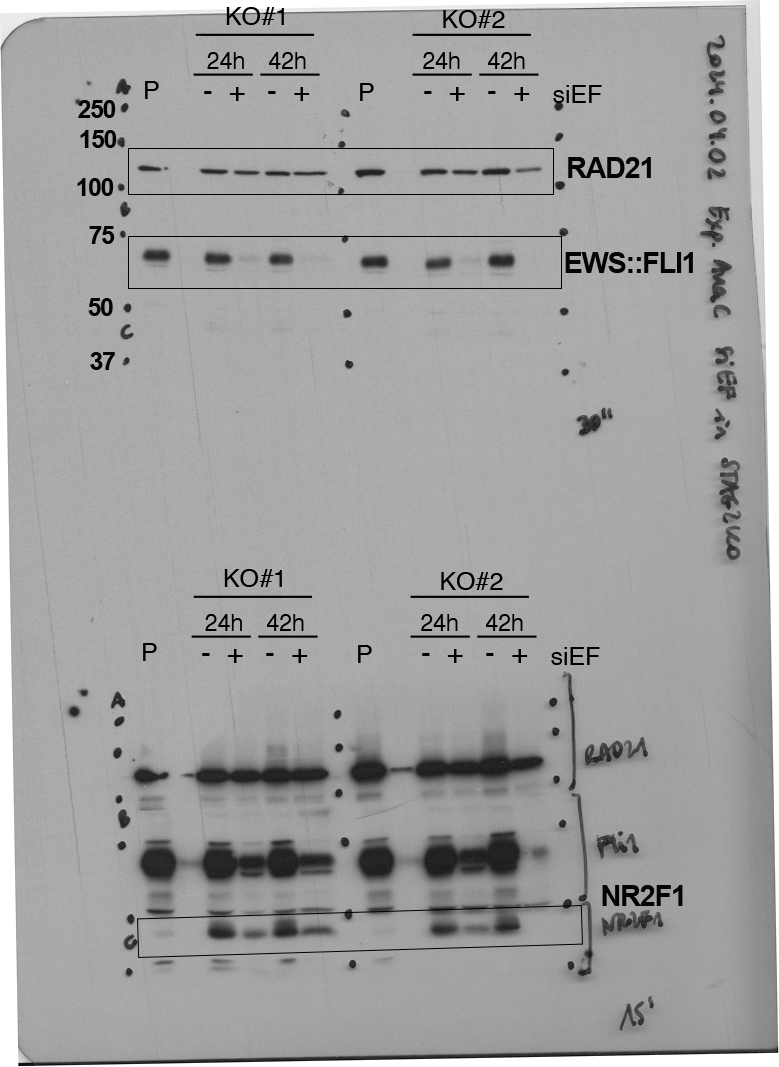

Supplement: Supplementary file 12 — Source data Fig. 5 [file 44319_2024_303_MOESM12_ESM.zip › 5B/western RAD21 EF NR2F1.tif]

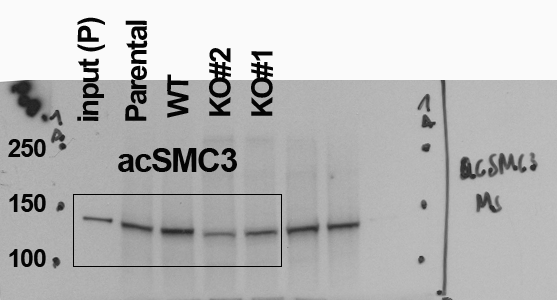

Supplement: Supplementary file 14 — Source data Fig. 7 [file 44319_2024_303_MOESM14_ESM.zip › 7B/western acSMC3.tif]

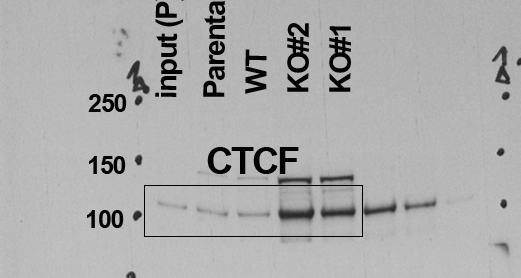

Supplement: Supplementary file 14 — Source data Fig. 7 [file 44319_2024_303_MOESM14_ESM.zip › 7B/western CTCF.tif]

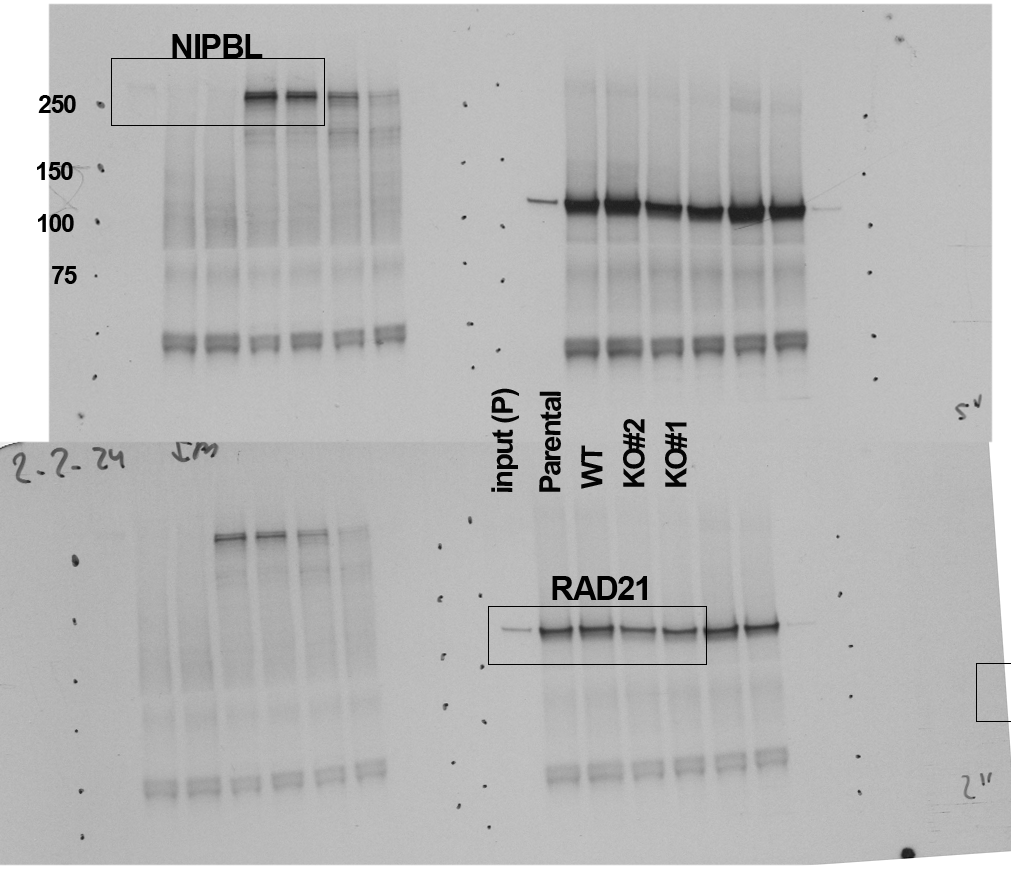

Supplement: Supplementary file 14 — Source data Fig. 7 [file 44319_2024_303_MOESM14_ESM.zip › 7B/western NIPBL RAD21.tif]

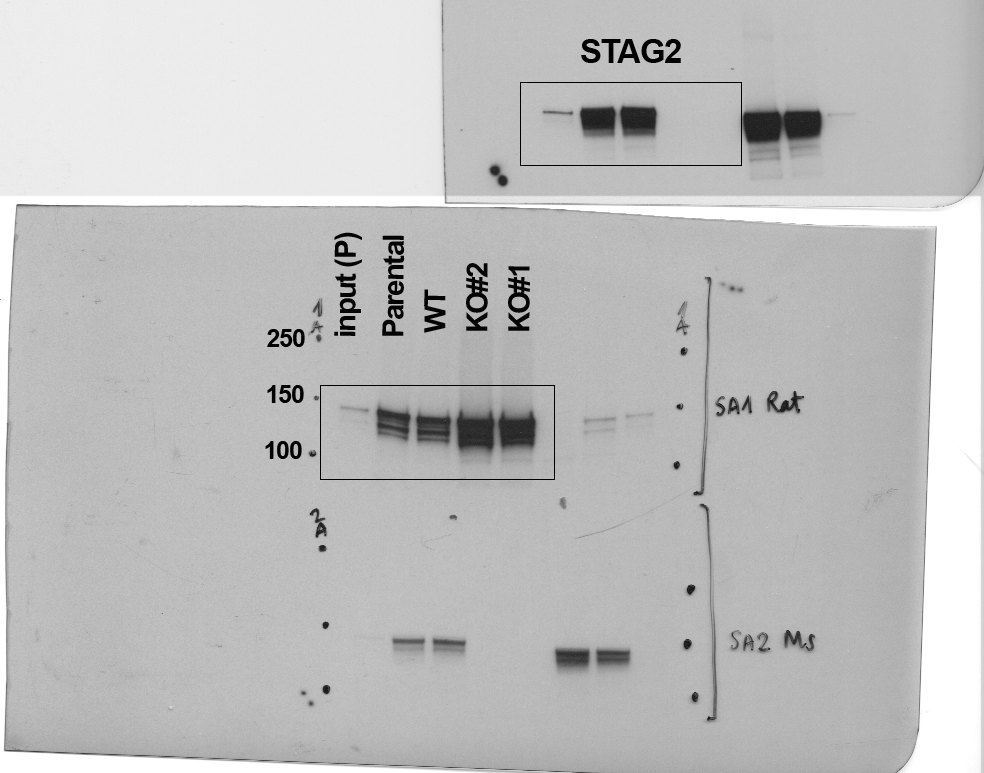

Supplement: Supplementary file 14 — Source data Fig. 7 [file 44319_2024_303_MOESM14_ESM.zip › 7B/western STAG1 STAG2.tif]

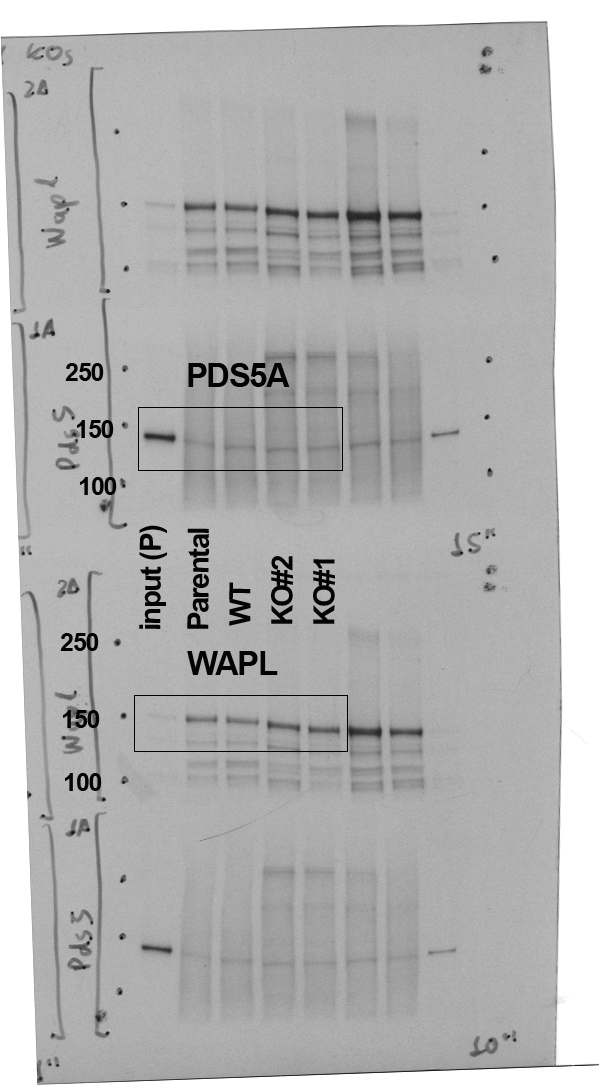

Supplement: Supplementary file 14 — Source data Fig. 7 [file 44319_2024_303_MOESM14_ESM.zip › 7B/western WAPL PDS5A.tif]
