# Supplementary material for: STAG2 loss in Ewing sarcoma alters enhancer-promoter contacts dependent and independent of EWS::FLI1
Source: EMBO Rep. 2024 Nov 1;25(12):5537–60. doi: 10.1038/s44319-024-00303-6 (PMC11624272; doi:10.1038/s44319-024-00303-6)
Supplement: Supplementary file 15 — Expanded View Figures [file 44319_2024_303_MOESM15_ESM.pdf]

Expanded View Figures

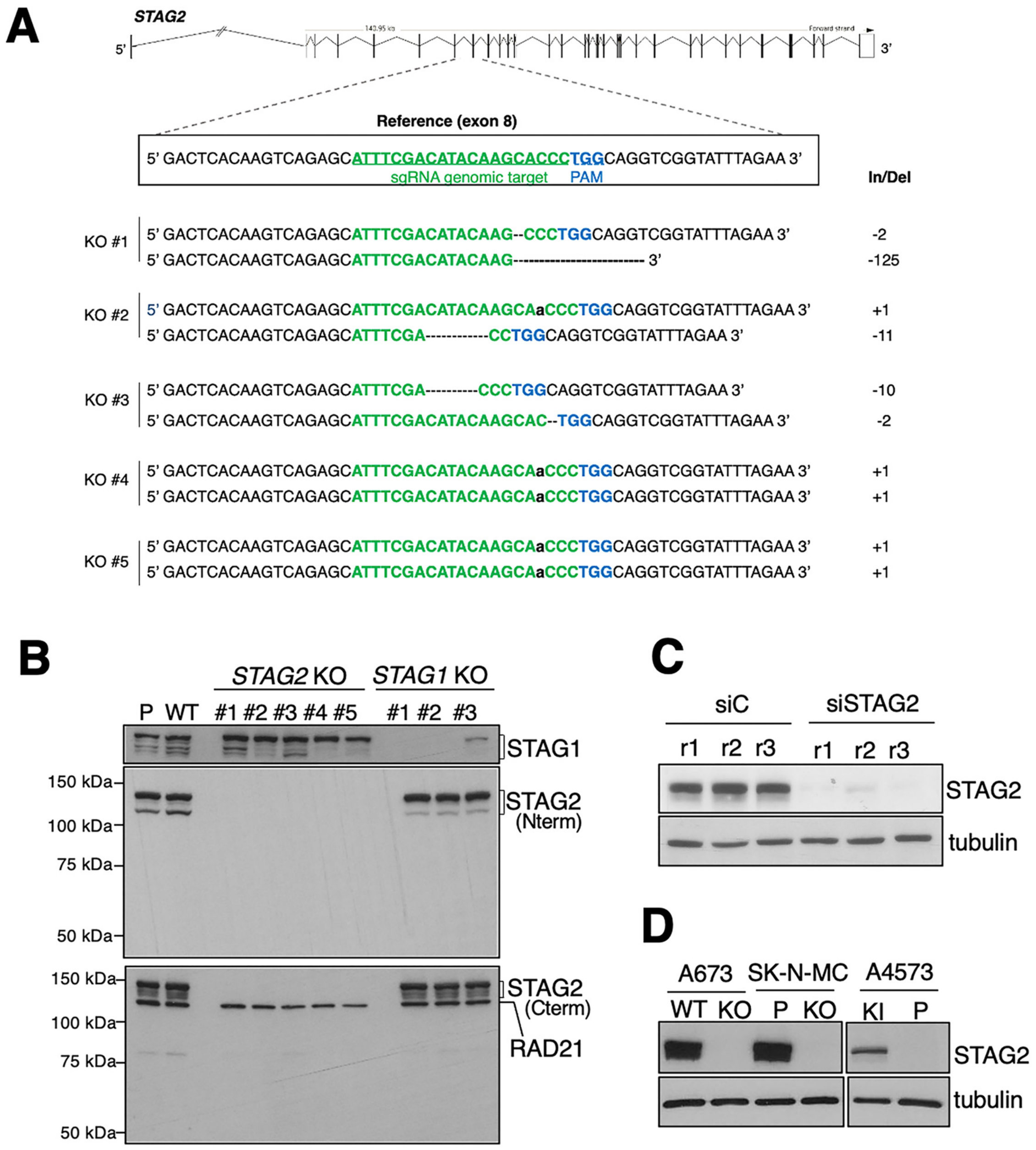

Figure EV1. Generation and characterization of Ewing sarcoma cell lines with and without STAG2.

(**A**) Mutations generated by CRISPR/Cas9 editing in *STAG2* gene. All clones were generated from a clone carrying an inducible Cas9 at the *AAVS1* locus (*STAG2* WT). (**B**) Immunoblot analysis of whole-cell extracts of parental (P) A673 cells and clones prepared in RIPA buffer. (**C**) A673 cells were mock transfected (control, siC) or transfected with siRNA against STAG2 (siSTAG2) and used for RNA-seq (3 replicates per condition, r1 to r3). Tubulin is used as loading control. (**D**) Immunoblot analysis of whole-cell extracts of Ewing sarcoma cells SK-N-MC and A4573 with and without STAG2, used for RNA-seq. A673 WT and *STAG2* KO#2 were used for comparison. Tubulin as loading control. Parental (P) SK-N-MC cells express STAG2 while A4573 cells do not, and they were edited to generate *STAG2* KO and KI clones, respectively, by CRISPR/Cas9 editing. Related to Fig. 1.

 © The Author(s)

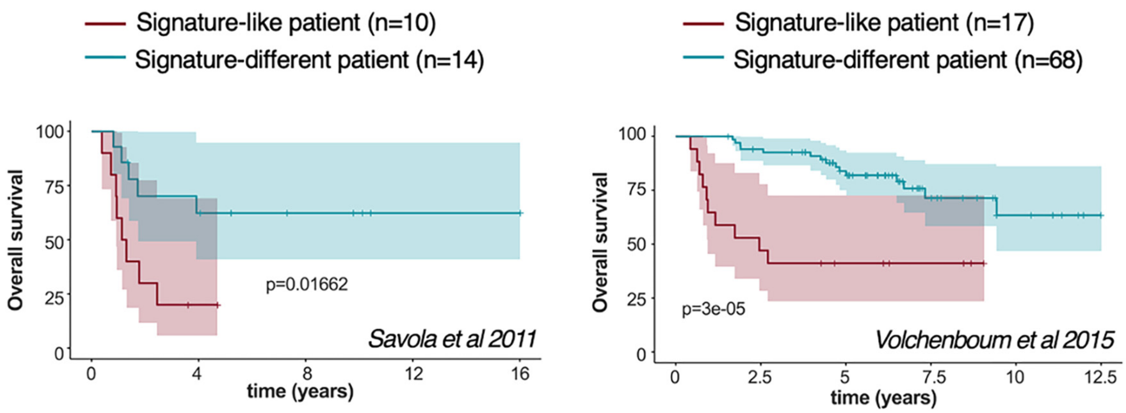

Figure EV2. Contribution of 232 STAG2-dependent genes to survival.

Overall survival probability (expressed as percentage) of patients from two different cohorts according to the survival signature described in main text. Only data from primary tumors were used. *P* values calculated with Cox proportional hazards regression. Related to Fig. 2.

© The Author(s)

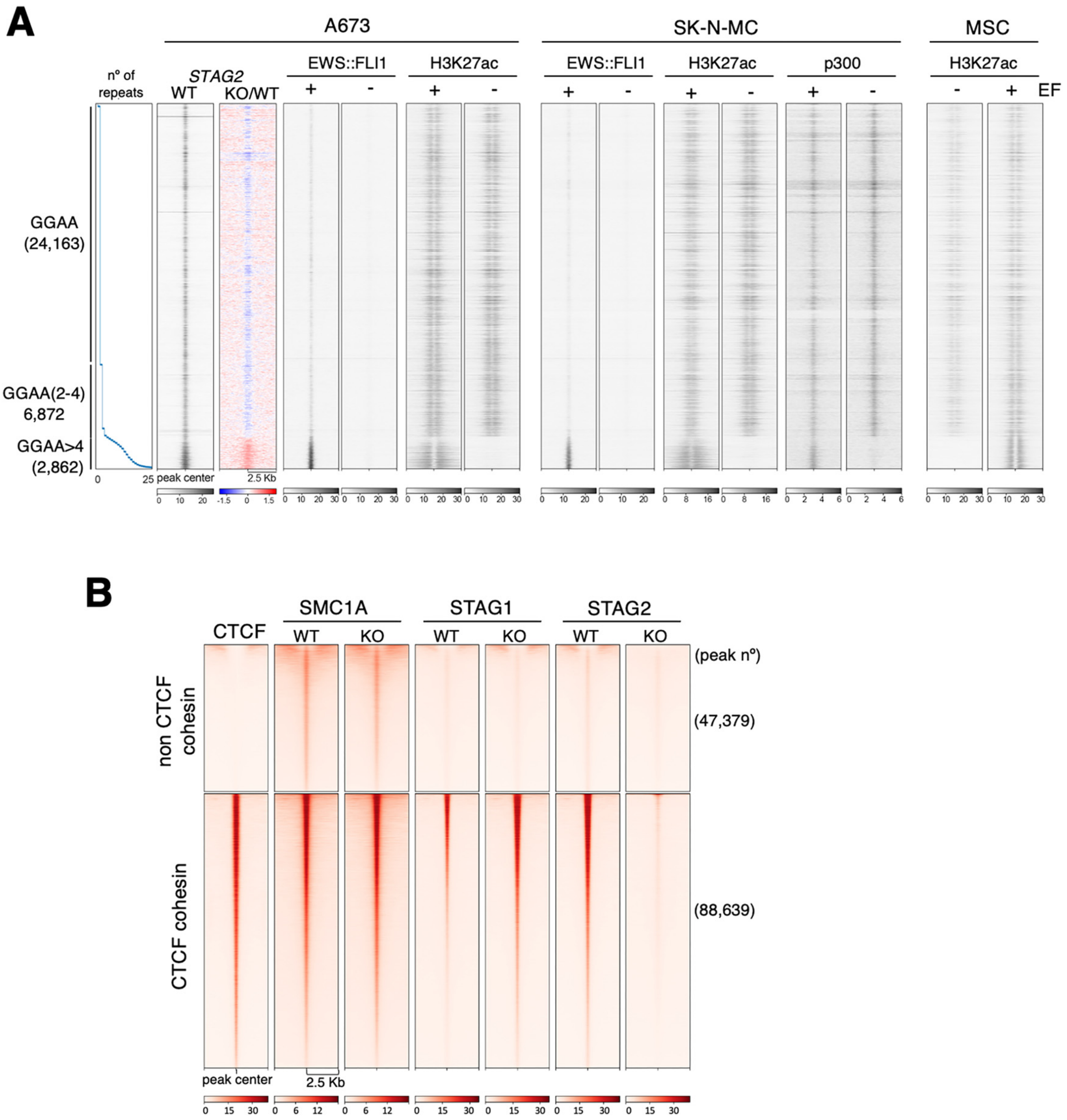

Figure EV3. Genomic profiling in Ewing sarcoma cell lines.

(**A**) Heatmaps showing EWS::FLI1 binding in A673 cells, ordered according to number of GGAA repeats. Peak calling was performed after merging data from two studies (Data ref: Surdez et al, 2021; Data ref: Adane et al, 2021). All other heatmaps showing occupancy of EWS::FLI1 (EF), H3K27ac and p300 around these peaks in A673 and SK-N-MC cells expressing shGFP (+EF) or shEF (−EF) as well as in mesenchymal stem cells (MSC) transfected with empty vector (−EF) or the oncogene (+EF), use data from (Data ref: Riggi et al, 2014). (**B**) Heatmaps showing ChIP-seq read distribution of cohesin in *STAG2* WT or *STAG2* KO A673 cells within a 5-kb window. Sites are clustered based on the presence of CTCF. Cohesin and CTCF data from (Data ref: Surdez et al, 2021) and (Data ref: Adane et al, 2021), respectively. Related to Fig. 3.

 © The Author(s)

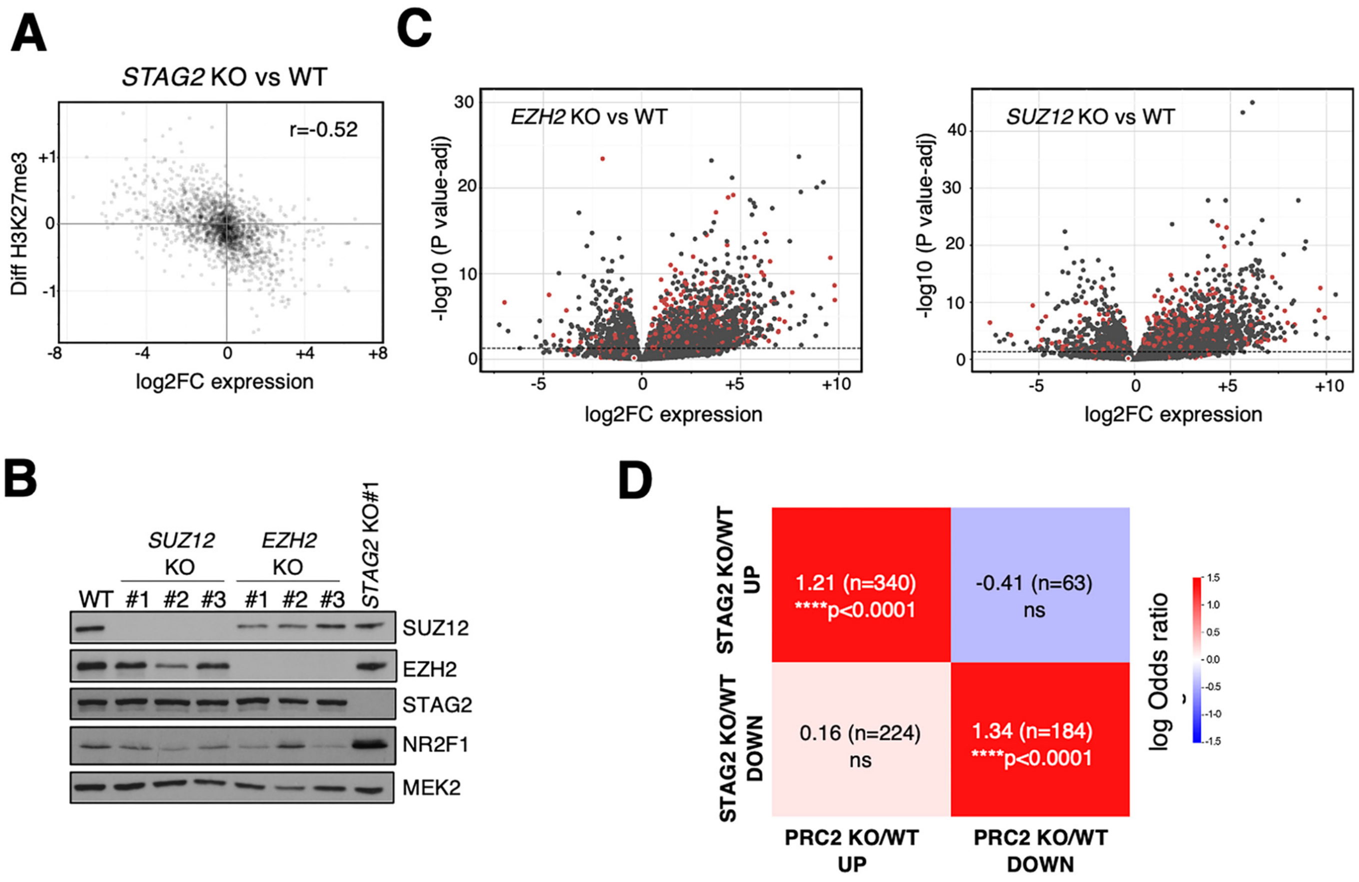

Figure EV4. Transcriptome similarities in STAG2 KO and PRC2 KO cells A673 cells.

(**A**) Scatterplot depicting changes in H3K27me3 at the promoter and gene expression in *STAG2* KO cells compared to STAG2 proficient cells. (**B**) Immunoblot analysis of whole-cell extracts of A673 clones KO for PRC2 components EZH2 and SUZ12. MEK2 serves as loading control. (**C**) Scatterplots showing differentially expressed genes in A673 clones KO for PRC2 components EZH2 and SUZ12. Genes deregulated also in *STAG2* KO cells (although not necessarily in the same direction) are colored in red. *P* values (*P* value-adj) were obtained using the DEseq2 package. (**D**) Comparison of genes significantly deregulated in PRC2 KO and *STAG2* KO A673 cells in the same direction. Chi-square test was applied. Related to Fig. 5.

A

Cohesin and regulators	log2FC (*STAG2* KO/WT)	FDR
NIPBL	0.176	0.145
MAU2	-0.032	0.869
WAPL	0.194	0.12
PDS5A	0.133	0.302
PDS5B	0.662	5.44E-13
ESCO1	0.283	0.319
ESCO2	0.445	0.332
HDAC8	0.229	0.046
CTCF	0.147	0.185
STAG1	0.412	0.036
STAG2	-1.946	2.84E-09
SMC1A	0.127	0.561
SMC3	0.092	0.492
RAD21	-0.085	0.595

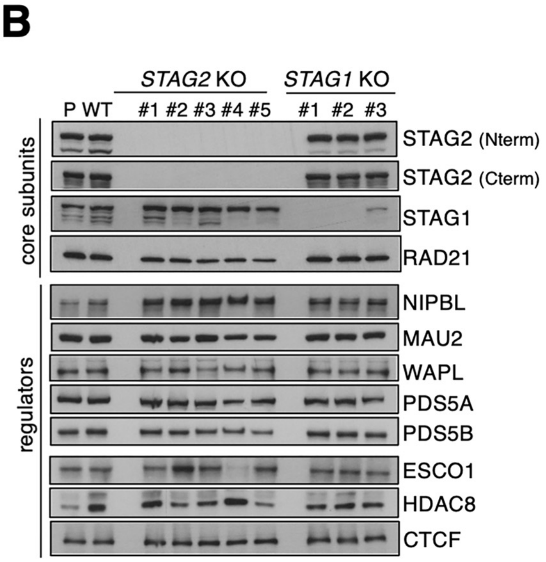

Figure EV5. Changes in gene expression and protein abundance of cohesin subunits and regulators in A673 cells with and without STAG2.

(**A**) Changes in gene expression levels of cohesin subunits and regulators assessed by RNA-seq of *STAG2* WT and KO A673 cells. Data taken from Dataset EV1A. Red and blue values correspond to significant up- and down-regulation, respectively (FDR < 0.05). (**B**) Immunoblot analysis of whole-cell extracts of parental (P) A673 cells and indicated clones prepared in RIPA buffer. Four gels were loaded to allow for immunoblotting with antibodies for cohesin subunits and regulators. The upper part of the figure is the same as in Fig. EV1B. Related to Fig. 7.

 © The Author(s)
